# Association between epidemiologic case definition categories and adverse clinical outcome in patients with *Clostridiodes difficile* infection in San Francisco County, California: a five-year retrospective cohort study

**DOI:** 10.1186/s12879-023-08030-4

**Published:** 2023-02-03

**Authors:** Bekure B. Siraw, Arthur L. Reingold, Didien Meyahnwi

**Affiliations:** 1 School of Public Health, University of California, 1279 Webster St. San Francisco, Berkeley, CA 94115 USA; 2 Division of Epidemiology, School of Public Health, University of California, Berkeley, CA USA

**Keywords:** *Clostridiodes difficile*, Adverse outcome, Mortality, Recurrence, Case definition category

## Abstract

**Background:**

Understanding the predictors of adverse clinical outcomes following incident *Clostridiodes difficile* infection (CDI) can help clinicians identify which patients are at risk of complications and help prioritize the provision of their care. In this study, we assessed the associations between epidemiologic case definition categories and adverse clinical outcomes in patients with CDI in San Francisco County, California.

**Methods:**

We conducted a retrospective cohort study using CDI surveillance data (n = 3274) from the California Emerging Infections Program for the time period 2016 to 2020. After independent associations were established, two multivariable logistic and log-binomial regression models were constructed for the final statistical analysis.

**Result:**

The mean cumulative incidence of CDI cases was 78.8 cases per 100,000 population. The overall recurrence rate and the 30-day all-cause mortality rate were 11.1% and 4.5%, respectively. After adjusting for potential confounders, compared to the community associated CDI cases, healthcare facility onset (AOR = 3.1; 95% CI [1.3–7]) and community-onset-healthcare facility associated (AOR = 2.4; 95% CI [1.4–4.3]) CDI cases were found to have higher odds of all-cause 30-day mortality. Community onset-healthcare facility-associated CDI case definition category was found to be significantly associated with an increased risk of recurrence of CDI (ARR = 1.7; 95% CI [1.2–2.4]).

**Conclusion:**

Although the incidence of community-associated CDI cases has been rising, the odds of all-cause 30-day mortality and the risk of recurrent CDI associated with these infections are lower than healthcare facility onset and community-onset healthcare facility-associated CDI cases.

## Background

*Clostridiodes difficile* (formerly *Clostridium difficile*) is an anaerobic gram-positive, spore-forming, toxin-producing bacillus that causes antibiotic-associated diarrhea or pseudomembranous colitis [1, 2]. Acquisition of *C. difficile*, most frequently through fecal–oral transmission, can result in asymptomatic colonization or clinical manifestations ranging from mild diarrhea to pseudomembranous colitis, bowel perforation, and death [3].

*C. difficile* is one of the five pathogens labeled as an urgent threat by the U.S. CDC [4]. It has produced a substantial burden on the US health care system for many years [5–7]. The economic implications of treating an incident CDI case have direct effects on healthcare facilities, third-party payers, and society as a whole [6]. By CDC’s estimate, in 2017 alone, there were 223,900 incident CDI cases and 12,800 deaths related to CDI, with over $1 billion in related healthcare costs [4]. Based on data from its ten active surveillance sites, CDC also estimated the crude annual incidence of CDI in the US in 2018 to be 65.9 cases per 100,000 population.

The CDC has been actively tracking incident CDI cases through its emerging infections program at ten surveillance sites, including The California Emerging Infection Program (CEIP) [8]. CEIP conducts surveillance, research, prevention, and control of emerging infectious diseases, including CDI. The CDI surveillance program at CEIP tracks the molecular, epidemiologic, and clinical trends in incident CDI cases in San Francisco County, California [9].

Although the incidence of CDI has been steadily falling in recent years, the proportion of CDI cases acquired in the community setting (community-associated CDI) has been increasing [7, 10]. Guh et al. estimated that the national burden of *C. difficile* infection was 476,400 cases in 2011 and 462,100 cases in 2017 [7]. After accounting for nucleic acid amplification test (NAAT) use, the adjusted estimate of the total burden of *C. difficile* infection decreased by 24% between 2011 and 2017; the adjusted estimate of the national burden of health care facility onset (HCFO) and community-onset healthcare facility associated (CO-HCFA) *C. difficile* infection decreased by 36%, whereas the adjusted estimate of the national burden of community-associated (CA) *C. difficile* infection increased slightly [7].

Adverse clinical outcomes in CDI patients can be represented by the incidence of clinical and laboratory indicators of severity, the rate of ICU admission, and the risk of mortality and recurrence [11–14]. Patients with these adverse outcomes are more challenging to treat, have longer hospital stays, and higher medical costs [6]. Accurate identification of factors that are associated with these adverse outcomes can have a substantial clinical and financial implication in the care of patients with CDI. Previous studies have identified various factors associated with all-cause mortality in CDI patients, including older age [15, 16], a WBC count > 15,000 [11, 16, 17], a serum creatinine level > 1.5 mg/dl, hypoalbuminemia [15, 16], a higher APACHE [11, 18] or qSOFA [15] score, prior corticosteroid use [11], and preexisting renal or pulmonary disease [11].

Reveles et al. reported that 17% of patients with an incident CDI experience recurrence [19]. Multiple factors have been reported to be associated with a higher risk of recurrent CDI, including older age [20, 21], higher serum C-reactive protein level [20], longer hospital stays for the initial CDI [20], a higher initial disease severity score [20] and the use of proton pump inhibitors [21].

In this study, we assessed the association between the epidemiologic case definition category of CDI cases (i.e., community-associated (CA), community-onset healthcare facility associated (CO-HCFA), and healthcare facility onset (HCFO)) and adverse clinical outcomes. We also assessed the trends in the incidence of the overall CDI cases and the three epidemiologic case definition categories of CDI in San Francisco between January 1st, 2016, and December 31st, 2020.

## Methods

### Study design

This was a retrospective cohort study of incident CDI cases in San Francisco between January 1st, 2016, and December 31st, 2020. Details of the data collection methods have been described previously [9]. Briefly, surveillance staff at the CEIP site identified all positive *C. difficile* test results from inpatient and outpatient laboratories serving residents in the surveillance area. Subsequently, a medical-record review was conducted to collect data on demographic characteristics, the location of stool sample collection, health care exposures, and outcomes in all cases of *C. difficile* infection [22]. Patients were followed for 12 months after the resolution of their initial CDI symptoms for the development of recurrent CDI.

### Inclusion and exclusion criteria

We used the CDI surveillance data from the California Emerging Infections Program. We included people age ≥ 18 years who had a positive stool toxin or molecular assay results for CDI between January 1st, 2016, and December 31st, 2020, and for whom the outcome and case definition category status were known. For the assessment of the risk factors for recurrence of CDI, we excluded cases with unknown recurrence status.

### Case definitions

A CDI case was defined as an individual with a stool specimen positive for *C. difficile* toxin by either enzyme immunoassay (EIA or GDH) or a molecular assay technique (NAAT). Cases that had a stool specimen positive for *C. difficile* toxin more than eight weeks after the last positive specimen were considered an incident case of CDI. CDI cases with a stool specimen positive for *C. difficile* within two to eight weeks of the last positive specimen were considered recurrent episodes of CDI. Epidemiologic case definition categories from CDC were used to categorize incident CDI cases into one of the three classes [8]: healthcare facility-onset (HCFO) if the initial specimen that yielded the positive *C. difficile* toxin result was collected more than three calendar days after admission to a healthcare facility; community-onset healthcare facility associated (CO-HCFA) if the initial specimen that yielded the positive *C. difficile* toxin result was collected in an outpatient setting or within the first 3 days of a healthcare facility admission and the patient had an overnight stay at a healthcare facility in the twelve weeks prior to the current presentation; and community-associated (CA) if the initial specimen that yielded the positive *C. difficile* toxin result was collected in an outpatient setting or within the first three days of a healthcare facility admission and the patient had no documented an overnight stay at a healthcare facility in the twelve weeks prior to the current presentation [8].

### Statistical analysis

Continuous variables were summarized using means and standard deviations and compared using a t-test. Categorical variables were summarized using proportions and compared using the chi-square test. We used bivariate logistic and log-linear regression to assess the level of significance of the associations between the predictors and the covariates with 30-day all-cause mortality and CDI recurrence rate, respectively. Using a p-value cutoff of 0.2, we subsequently included the variables that had statistically significant associations with the outcomes in the two final multivariate logistic and log-linear regression models for the assessment of 30-day all-cause mortality and CDI recurrence, respectively. Because the mortality rate in our cohort was 4.5%, the rare disease assumption was made and the odds ratio outputs of the logistic regression model were assumed to approximate the relative risk of mortality. Because there were 333 (10.17%) and 107 (7.23%) observations that were second positive samples from the same subject in the final analysis for CDI outcome and recurrence respectively, we performed a sensitivity analysis by excluding these observations, and redoing the bivariate and multivariate regression analysis. However, these showed no significant changes in the significance or strength of associations we found in both of our outcome variables without the exclusion of the observations. The output of all tests was reported with a 95% confidence interval and a 0.05 level of significance. All the data cleaning, manipulation, visualization, and statistical analysis were done using the statistical software R (version 4.1.2).

## Results

### Study population

A total of 3274 patients fulfilling the CDI case definition criteria diagnosed between January 1, 2016, and December 31, 2020 were included. The median age of patients in the cohort was 66 years (interquartile range (IQR) 52–78). 1760 (54%) were men and 335(11%) were of Hispanic/Latino ethnicity. Whites (39.7%) and Asians (38.2%) were the predominant races in the cohort, with Blacks and other races accounting for 8.5% and 15.6% of the cohort, respectively. CDI cases were classified as healthcare facility associated (HCFA) in 1177 (36.2%), community-onset healthcare facility associated (CO-HCFA) in 768 (23.6%), and community-associated (CA) in 1310 (40.2%). Table 1 presents a summary of the distribution of socio-demographic factors in the CDI cases, broken out by case definition categories. 2384 (72.8%), 890 (27.2%), and 1393 (42.5%) of the patients had a positive NAAT, toxin EIA, and GDH results, respectively. Excluding the patients who have a positive NAAT test result, the remaining patients were positive for both toxin EIA and GDH. There was no significant association found between the diagnostic methods used and risk of CDI recurrence or mortality.

**Table 1:** Demographic characteristics of patients with *Clostridiodes difficile* infection in San Francisco County, California from 2016 to 2020

	Overall	CO	HCFO	CO-HCFA
n	3274	1322	1180	772
Age (%)				
< 45 years	593 (18.2)	361 (27.4)	126 (10.7)	106 (13.8)
45–65 years	1017 (31.2)	401 (30.5)	338 (28.7)	278 (36.2)
65–85 years	1222 (37.5)	429 (32.6)	508 (43.1)	285 (37.1)
> 85 years	431 (13.2)	125 (9.5)	206 (17.5)	100 (13.0)
Ethnicity (%)				
Hispanic	356 (10.9)	137 (10.4)	133 (11.3)	86 (11.1)
Non-Hispanic	2211 (67.5)	788 (59.6)	887 (75.2)	536 (69.4)
Unknown	707 (21.6)	397 (30.0)	160 (13.6)	150 (19.4)
Race (%)				
White	1298 (39.6)	538 (40.7)	442 (37.5)	318 (41.2)
Black	421 (12.9)	118 (8.9)	177 (15.0)	126 (16.3)
Asian	859 (26.2)	316 (23.9)	357 (30.3)	186 (24.1)
American Indian	8 (0.2)	5 (0.4)	1 (0.1)	2 (0.3)
Pacific Islander	28 (0.9)	12 (0.9)	5 (0.4)	11 (1.4)
Unknown	660 (20.2)	333 (25.2)	198 (16.8)	129 (16.7)
Sex (%)				
Female	1508 (46.1)	673 (50.9)	482 (40.8)	353 (45.7)
Male	1766 (53.9)	649 (49.1)	698 (59.2)	419 (54.3)

*HCFO *Healthcare Facility Onset, *CO-HCFA* Community-Onset Healthcare Facility Associated, *CA* Community Associated

### Medical conditions, clinical features, and previous medication exposures

Diabetes mellitus, chronic kidney disease, and chronic obstructive pulmonary disease were the three most prevalent conditions, present in 15.6%, 14.7%, and 9.6% of patients in the cohort, respectively. Table 2 summarizes the frequency distribution of selected medical conditions. All 3274 patient had documented diarrhea. 2702 (82.5%) had > 3 episodes of diarrhea per day for > 1 day whereas 572 (17.5) had diarrhea with undocumented frequency or duration.

**Table 2:** Frequency distribution of medical comorbidities across the epidemiologic CDI case definition categories in San Francisco County, California, 2016–2020

	Overall	CA	HCFO	CO-HCFA
n	3274	1322	1180	772
Liver disease (%)	174 (5.3)	71 (5.4)	10 (0.8)	93 (12.0)
AIDS (%)	45 (1.4)	24 (1.8)	1 (0.1)	20 (2.6)
HIV (%)	104 (3.2)	52 (3.9)	4 (0.3)	48 (6.2)
Heart failure (%)	228 (7.0)	72 (5.4)	22 (1.9)	134 (17.4)
Hematologic malignancy (%)	62 (1.9)	26 (2.0)	4 (0.3)	32 (4.1)
Non metastatic solid organ tumor (%)	179 (5.5)	94 (7.1)	13 (1.1)	72 (9.3)
Metastatic solid organ tumor (%)	112 (3.4)	45 (3.4)	8 (0.7)	59 (7.6)
Organ transplant recipient (%)	46 (1.4)	21 (1.6)	3 (0.3)	22 (2.8)
Stem cell transplant recipient (%)	12 (0.4)	6 (0.5)	0 (0.0)	6 (0.8)
Diverticulosis (%)	150 (4.6)	89 (6.7)	2 (0.2)	59 (7.6)
Inflammatory bowel disease (%)	134 (4.1)	103 (7.8)	2 (0.2)	29 (3.8)
CKD (%)	473 (14.4)	221 (16.7)	28 (2.4)	224 (29.0)
COPD (%)	303 (9.3)	162 (12.3)	13 (1.1)	128 (16.6)
Diabetes (%)	505 (15.4)	233 (17.6)	39 (3.3)	233 (30.2)
Total	3274	1322 (40.4)	1180 (36)	772 (23.5)

*HCFO* Healthcare Facility Onset, *CO-HCFA* Community-Onset Healthcare Facility Associated, *CA* Community Associated, *HIV* Human Immunodeficiency Virus, *AIDS* Acquired Immunodeficiency Syndrome, *COPD* Chronic Obstructive Pulmonary Disease, *CKD* chronic kidney disease

Nausea, and vomiting, observed in 7.7%, and 5.7% of the cases, were the most frequent additional clinical presentations. In our assessment of exposure to medications within 12 weeks before the incident CDI, proton pump inhibitors (21%) were the medications most often identified. Vancomycin (10.6%) and ceftriaxone (10.1%) were the two most frequent antibiotic exposures.

**Fig. 1:**
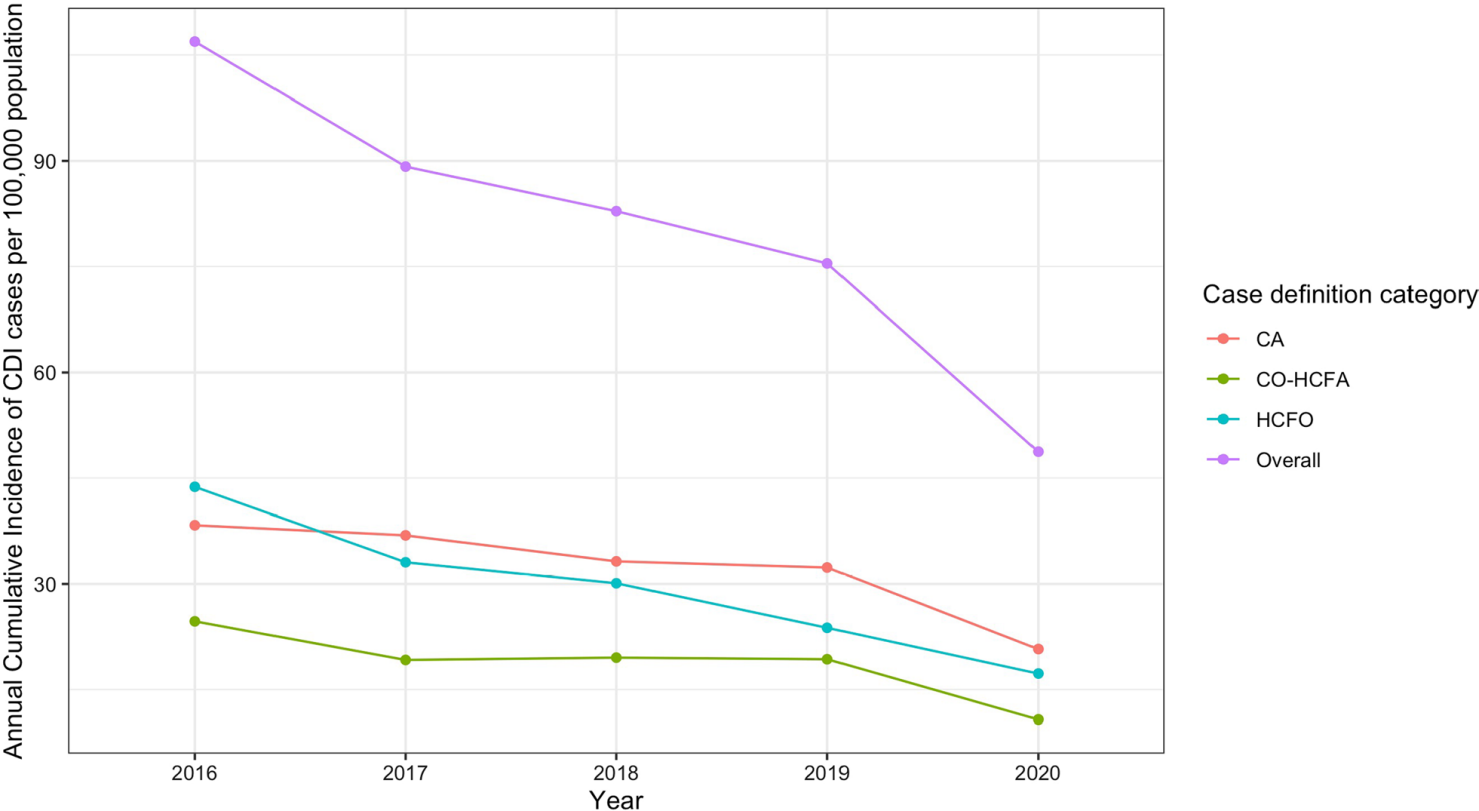
The annual cumulative incidence of overall Clostrdiodes Difficile cases and the three case definition categories in San Francisco County from 2016 to 2020

The 5-year mean cumulative incidence of CDI cases per 100,000 population was 78.2. The five-year mean case definition category-specific cumulative incidences per 100,000 for HCFO, CO-HCFA, and CA CDI were 27.6, 17.1, and 32, respectively. Figure 1 shows the trends in annual overall and CDI case definition category-specific cumulative incidence throughout the study period. There were 150 deaths within 30 days of admission, yielding a total all-cause 30-day mortality of 4.6%. Of the 150 deaths HCFO, CO-HCFA, and CA cases account for 60.7%, 24.7%, and 14.6%, respectively. The rate of recurrent CDI cases was 11.1%. HCFO, CO-HCFA, and CA cases account for 4.9%, 46.9%, and 48.2% of recurrent cases, respectively.

### Results of bivariate analysis for independent associations with unadjusted odds ratios

#### All-cause 30-day mortality

In the bivariate analysis, we found several variables to be independently associated with increased odds of mortality. Compared to CA CDI, HCFO (OR = 7.4; 95% CI [4.7–12]) and CO-HCFA (OR = 3.6; 95% CI [2.2–6.2]) CDIs were both significantly associated with a higher odds of mortality. Compared to those 18 to 44 years of age, the odds of mortality were higher in those 45 to 64 (OR = 4.8; 95% CI [2.3–11.7]), 65–84 (OR = 6.4; 95% CI [3.2–15.3]), and ≥ 85 (OR = 10.3; 95% CI [4.9–25.2]) years of age. A white blood cell count > 15,000 cells/mm^3^ and a serum albumin concentration < 2.5 mg/dl were also each associated with a higher odds of mortality with odds ratios of 2.2 (95%CI [1.4–3.6]) and 3.5 (95%CI [2–7]), respectively. Male sex was associated with a higher odds of mortality with an OR of 1.34 (95% CI [1.002–1.8]. Compared to Whites, Asians had higher odds of mortality, with an OR of 1.58 (95% CI [1.13–2.2]. Exposure to clindamycin (OR = 3.4; 95% CI 1.3–13.7) and to metronidazole (OR = 3.4; 95% CI [1.4–11.2]) in the 12 weeks before the incident CDI episode were each independently associated with an increase in the odds of mortality, as was ICU admission (OR = 4; 95% CI [2.6–6]). The presence of a hematologic malignancy was the only medical condition that was significantly associated with a higher odds of mortality (OR = 2.4 (95% CI [1.03–4.8]).

#### Recurrence of CDI

In the bivariate analysis, we found no statistically significant difference in the relative risk of CDI recurrence between the CA and HCFO cases categories. However, compared to CA cases, CO-HCFA cases had a higher risk of CDI recurrence with an RR of 1.78 (95% CI [1.3–2.5]). Compared to those 18 to 44 years of age, only those ≥ 85 years of age had a higher risk of CDI recurrence (RR = 2.2; 95% CI [1.25–4]).

### Results of the multivariate logistic regression models with adjusted OR

#### All-cause 30-day mortality

In the multivariate logistic regression model, we found that HCFO (AOR = 3.1; 95% CI [1.3–7]) and CO-HCFA (AOR = 2.4; 95% CI [1.4–4.3]) CDI cases had a significantly increased odds of mortality, compared to CA CDI cases. Male sex and recent exposure to clindamycin and metronidazole were not significantly associated with increased odds of mortality in the adjusted model (Table 3).

**Table 3:** Multivariate logistic regression model output with adjusted odds ratio of predictors of CDI-associated mortality

	Adjusted OR^1^	95% CI^1^	p-value
Case definition category			
CA	Reference		
HCFO	3.07	1.29, 6.95	0.008
CO-HCFA	2.40	1.37, 4.33	0.003
Age			
18–44 years	Reference		
45–64 years	2.44	0.80, 10.6	0.2
65–84 years	2.96	1.00, 12.7	0.083
> 85 years	6.31	2.00, 28.0	0.005
WBC < 15,000 cells/mm^3^	3.00	1.77, 5.15	< 0.001
Serum Albumin < 2.5 mg/dl	3.92	2.09, 7.91	< 0.001
Hematologic malignancy	4.58	1.69, 11.3	0.002
Race			
White	Reference		
Asian	1.80	1.02, 3.19	0.041
ICU admission	5.01	2.82, 8.82	< 0.001

^a^*OR* odds ratio, *CI* confidence interval, *HCFO* Healthcare Facility Onset, *CO-HCFA* Community-Onset Healthcare Facility Associated, *CA* Community Associated, *WBC* white blood cells

#### Recurrence of CDI

In the adjusted multivariate log-binomial regression model, we found that CO-HCFA and age ≥ 85 years were each significantly associated with an increased risk of recurrence, with adjusted relative risks of 1.7 (95% CI [1.2–2.4]) and 2 (95% CI [1.1–3.7]), respectively. None of the medical conditions, laboratory parameters, or ICU admission status were significantly associated with an increased risk of recurrence of CDI.

## Discussion

Our results shed light on the recent changes in the epidemiologic trends and prognostic implications of the three epidemiologic case definition categories of *Clostridiodes difficile* infection. To our knowledge, this is the first study assessing the association between the three CDI case definition categories and adverse clinical outcomes, including mortality and recurrence of CDI.

The five-year mean cumulative incidence of CDI cases in our study population was 78.2 per 100,000, a rate comparable to the cumulative incidence reported in prior studies [23, 24]. Compared to the previous years, the overall annual cumulative incidence of CDI cases dropped significantly in the year 2020. The increased focus on hand hygiene, environmental cleaning, patient isolation, and use of PPE in response to the SARS CoV-2 pandemic during 2020, combined with continued inpatient antimicrobial stewardship programs and a marked decline in outpatient antibiotic prescribing, may have contributed to the reduction in the cumulative incidence of CDI during 2020 compared to previous years [25].

The all-cause 30-day mortality rates and the CDI recurrence rates reported in previous studies ranged between 8–15% [26–28] and 17–23% [19, 20, 23], respectively which are slightly higher than the rates we found. This difference may be attributable to our cohort being relatively younger and the lower incidence of comorbid medical condition in our cohort compared to the other studies. In addition to this the recent changes in the Infectious Diseases Society of America (IDSA) and Society for Healthcare Epidemiology of America (SHEA) CDI treatment guidelines leading to an increase in the usage of oral vancomycin in the treatment of CDI in recent years has also likely played a role in this finding [3].

We were not able to identify prior studies assessing the association between epidemiologic case definition categories and the risk of mortality and CDI recurrence. We found that, compared to CA CDI cases, both HCFO and CO-HCFA CDI cases were associated with an increased odds of all-cause mortality in 30 days. Although residual confounding cannot be fully excluded, after adjusting for multiple confounders, including, age, race, sex, comorbid medical conditions, medication use, and severity of the incident CDI, the adjusted odds ratios were found to be significantly elevated. This finding has prognostic implications and helps clinicians and public health personnel in prioritizing areas of intervention in the prevention, treatment, and surveillance of CDI. One potential explanation for this could be differences in the *C. difficile* strains causing the three types of infections. This requires further genetic and molecular analysis, which is beyond the scope of this study.

We found that the relative risk of a CDI recurrence was higher among CO-HCFA CDI cases compared to the CA CDI cases. There was no statistically significant difference in the risk of CDI recurrence between CA and HCFO CDI cases. This may be because of the very low frequency of CDI recurrences in those with HCFO CDI, compared to the other categories in our cohort. Although several prior studies have reported an increased risk of CDI recurrence is associated with low serum albumin [29], proton pump inhibitor use [20, 21, 29], ICU admission [30], hematologic malignancy [31], and male sex [32], our analysis didn’t show statistical significance for any of these variables.

Medical conditions such as heart failure [33], chronic obstructive pulmonary disease [16], chronic kidney disease12,34, and diabetes melllitus [26] have previously been associated with an increased odds of all-cause mortality among CDI patients. In our cohort, after adjusting for multiple confounders, the only comorbid medical condition that was found to be significantly associated with an increased odds of mortality was the presence of a hematologic malignancy [31].

Keneally et al. previously reported an increased odds of mortality in CDI patients who had been admitted to the ICU [18]. We found a similar association between ICU admission and the odds of mortality in CDI patients.

We acknowledge that our study was subject to several limitations. First, because we were doing secondary data analysis, we were not able to identify the causes of death for the patients who died. Using all-cause mortality instead of cause-specific mortality as an outcome measure may have overestimated the odds ratio estimates reported in this paper. Second, more than half of the patients in our dataset had an unknown CDI recurrence status. In our CDI recurrence analysis, we excluded these patients with unknown recurrence statuses, which may have biased our risk ratio estimates in an unknown direction, as these patients may be significantly different from those with a known recurrence status. Third, there were only eight cases of recurrent CDI in the HCFO category, a number that is very low compared to the other case definition categories. With HCFO being found to have higher odds of 30 day all-cause mortality, the lower number of recurrences found in this case definition category may be due to competing risk of mortality. In our final analysis, this may have contributed to the non-significant risk ratio estimate found when comparisons were made in the recurrent CDI cases between the CA and HCFO CDI cases. However, the time of death is not consistently recorded across dataset to perform the time to event analysis and assess for competing risks.

## Conclusion

*Clostridiodes difficile* is one of five pathogens labeled as an urgent threat by the CDC. The infection it causes affects a subgroup of high-risk patients and often leads to a variety of complications. Studies have reported multiple factors that predict mortality in CDI patients. In this study, we found that the HCFO and CO-HCFA CDI case definition categories were significantly associated with an increased odds of mortality in these patients. We also found that compared to CA CDI cases, CO-HCFA cases have a higher risk of recurrent *C. difficile* infection. Accurate identification of such risk factors in CDI patients would help clinicians anticipate the complications and prioritize the delivery of care based on need, thereby maximizing the impacts of these interventions.

## Data Availability

All data analyzed during this study are included in the Additional files.

## Abbreviations

CDI: *Clostridiodes difficile* Infection
AOR: Adjusted odds ratio
ARR: Adjusted relative risk
CDC: Center for disease control
CEIP: California emerging infections program
NAAT: Nucleic acid amplification test
HCFO: Healthcare facility onset
CO-HCFA: Community onset-healthcare facility onset
CA: Community associated
APACHE: Acute physiology and chronic health evaluation
qSOFA: Quick sequential organ failure assessment
EIA: Enzyme immunoassay
GDH: Glutamate dehydrogenase
PPE: Personal protective equipment
SARS CoV-2: Severe acute respiratory syndrome corona virus 2
ICU: Intensive care unit

## References

[1] Leffler DA, Lamont JT (2015). *Clostridium difficile* infection. N Engl J Med.

[2] Bartlett JG, Chang TW, Gurwith M, Gorbach SL, Onderdonk AB (1978). Antibiotic-associated pseudomembranous colitis due to toxin-producing clostridia. N Engl J Med.

[3] McDonald LC, Gerding DN, Johnson S (2018). Clinical Practice Guidelines for *Clostridium difficile* Infection in Adults and Children: 2017 Update by the Infectious Diseases Society of America (IDSA) and Society for Healthcare Epidemiology of America (SHEA). Clin Infect Dis.

[4] Antibiotic resistance threats in the United States, 2019. 2019; Available at: https://stacks.cdc.gov/view/cdc/82532. Accessed 6 Feb 2022.

[5] Dubberke ER, Olsen MA (2012). Burden of *Clostridium difficile* on the Healthcare System. Clin Infect Dis.

[6] McGlone SM, Bailey RR, Zimmer SM (2012). The economic burden of *Clostridium difficile*. Clin Microbiol Infect.

[7] Guh AY, Mu Y, Winston LG (2020). Trends in U.S. burden of *Clostridiodes difficile* infection and outcomes. N Eng J Med..

[8] *Clostridiodes difficile* Infection (CDI) Tracking|HAIC Activities|HAI|CDC. Available at: https://www.cdc.gov/hai/eip/cdiff-tracking.html. Accessed 4 Feb 2022.

[9] *Clostridium difficile* Infection|CEIP. Available at: https://ceip.us/projects/haic/clostridium-difficile-infection-surveillance/. Accessed 4 Feb 2022.

[10] Turner NA, Grambow SC, Woods CW (2019). Epidemiologic Trends in *Clostridiodes difficile* Infections in a Regional Community Hospital Network. JAMA Netw Open.

[11] Dudukgian H, Sie E, Gonzalez-Ruiz C, Etzioni DA, Kaiser AMC (2010). difficile colitis–predictors of fatal outcome. J Gastrointest Surg.

[12] Cadena J, Thompson GR, Patterson JE (2010). Clinical predictors and risk factors for relapsing *Clostridium difficile* infection. Am J Med Sci.

[13] Bloomfield MG, Carmichael AJ, Gkrania-Klotsas E (2013). Mortality in *Clostridium difficile* infection: a prospective analysis of risk predictors. Eur J Gastroenterol Hepatol.

[14] Pant C, Madonia P, Minocha A, Manas K, Jordan P, Bass P (2010). Laboratory markers as predictors of mortality in patients with *Clostridium difficile* infection. J Investig Med.

[15] Marra AR, Edmond MB, Wenzel RP, Bearman GM (2007). Hospital-acquired *Clostridium difficile*-associated disease in the intensive care unit setting: epidemiology, clinical course and outcome. BMC Infect Dis.

[16] Sailhamer EA, Carson K, Chang Y (2009). Fulminant *Clostridium difficile* colitis: patterns of care and predictors of mortality. Arch Surg.

[17] Bishara J, Peled N, Pitlik S, Samra Z (2008). Mortality of patients with antibiotic-associated diarrhoea: the impact of *Clostridium difficile*. J Hosp Infect.

[18] Kenneally C, Rosini JM, Skrupky LP (2007). Analysis of 30-day mortality for *Clostridium difficile*-associated disease in the ICU setting. Chest.

[19] Reveles KR, Lawson KA, Mortensen EM (2017). National epidemiology of initial and recurrent *Clostridium difficile* infection in the Veterans Health Administration from 2003 to 2014. PLoS ONE.

[20] Eyre DW, Walker AS, Wyllie D (2012). Predictors of first recurrence of *Clostridium difficile* infection: implications for initial management. Clin Infect Dis.

[21] Lupse M, Mirela F, Cioara A, Flonta M, Filipescu I, Todor N (2013). Predictors of first recurrence in Clostridium diicile-associated disease. A study of 306 patients hospitalized in a Romanian Tertiary Referral Center. J Gastrointestin Liver Dis.

[22] Lessa FC, Mu Y, Winston LG (2014). Determinants of *Clostridium difficile* infection incidence across diverse United States geographic locations. Open Forum Infect Dis.

[23] Lübbert C, Zimmermann L, Borchert J, Hörner B, Mutters R, Rodloff AC (2016). Epidemiology and recurrence rates of *Clostridium difficile* infections in Germany: a secondary data analysis. Infect Dis Ther.

[24] Khanna S, Gupta A, Baddour LM, Pardi DS (2016). Epidemiology, outcomes, and predictors of mortality in hospitalized adults with *Clostridium difficile* infection. Intern Emerg Med.

[25] Weiner-Lastinger LM, Pattabiraman V, Konnor RY (2022). The impact of coronavirus disease 2019 (COVID-19) on healthcare-associated infections in 2020: a summary of data reported to the National Healthcare Safety Network. Infect Control Hosp Epidemiol.

[26] Honda H, Yamazaki A, Sato Y, Dubberke ER (2014). Incidence and mortality associated with *Clostridium difficile* infection at a Japanese tertiary care center. Anaerobe.

[27] Hensgens MPM, Goorhuis A, Dekkers OM, van Benthem BHB, Kuijper EJ (2013). All-cause and disease-specific mortality in hospitalized patients with *Clostridium difficile* infection: a multicenter cohort study. Clin Infect Dis.

[28] Kassam Z, Cribb Fabersunne C, Smith MB (2016). *Clostridium difficile* associated risk of death score (CARDS): a novel severity score to predict mortality among hospitalised patients with *C. difficile* infection. Aliment Pharmacol Ther.

[29] Kim JW, Lee KL, Jeong JB (2010). Proton pump inhibitors as a risk factor for recurrence of Clostridium-difficile-associated diarrhoea. World J Gastroenterol.

[30] Jasiak NM, Alaniz C, Rao K, Veltman K, Nagel JL (2016). Recurrent *Clostridium difficile* infection in intensive care unit patients. Am J Infect Control.

[31] Scappaticci GB, Perissinotti AJ, Nagel JL, Bixby DL, Marini BL (2017). Risk factors and impact of *Clostridium difficile* recurrence on haematology patients. J Antimicrob Chemothera.

[32] Fekety R, McFarland LV, Surawicz CM, Greenberg RN, Elmer GW, Mulligan ME (1997). Recurrent *Clostridium difficile* diarrhoea: characteristics of and risk factors for patients enrolled in a prospective, randomized, double-blinded trial. Clin Infect Dis.

[33] Wilson V, Cheek L, Satta G (2010). Predictors of death after *Clostridium difficile* infection: a report on 128 strain-typed cases from a teaching hospital in the United Kingdom. Clin Infect Dis.

